# Knowledge of Human Papillomavirus, Risk of Anogenital Cancers, and Benefits of Vaccination: A Medical Student Survey in Saudi Arabia

**DOI:** 10.7759/cureus.5051

**Published:** 2019-07-01

**Authors:** Salman N Almutairi, Abullah A Aljalfan, Zuhour A Alqahtani, Asem M Shadid, Salah-Ud-Din Khan

**Affiliations:** 1 Miscellaneous, Imam Mohammad Ibn Saud Islamic University, Riyadh, SAU; 2 Miscellaneous, Imam Muhammed Ibn Saud Islamic University, Riyadh, SAU; 3 Biochemistry, College of Medicine, Imam Mohammad Ibn Saud Islamic University, Riyadh , SAU

**Keywords:** hpv, cancer, saudi arabia, medical students, vaccination

## Abstract

Background

Human papillomavirus (HPV) is one of the most common sexually transmitted infections and plays a significant role in the development of anogenital cancer. However, there is a lack of awareness on the subject in Saudi Arabia and very few documented studies on the knowledge and awareness of medical students regarding HPV.

Methods

A cross-sectional study utilizing a survey distributed to 306 medical students of both genders was utilized. A self-administrated questionnaire was distributed to all participants to assess their knowledge of HPV and their attitude towards HPV vaccination. Data analysis was performed using SPSS v 24 (IBM Corp., Armonk, NY, US) and RStudio v 1.14 (Boston, US).

Results

Most of the respondents (72.2%) had a high level of knowledge regarding HPV, with 47.84% of the respondents being aware of the risks associated with HPV and having a positive attitude towards HPV. Female respondents had better awareness and knowledge as compared to male students. However, there was a lack of knowledge regarding the duration and frequency of HPV infection, which could have an effect on the attitude of medical students towards vaccination. The results obtained by using Pearson’s correlation coefficient showed a statistically significant correlation between the attitude towards HPV and the knowledge regarding HPV (p-value < 0.05).

Conclusion

Better awareness and knowledge regarding HPV and its risks correlate with a better attitude towards HPV. The medical school curriculum was found to be a primary source of information for students on the awareness of HPV. Therefore, more about HPV and the benefits associated with vaccination against HPV should be included in the curriculum in all years of medical school.

## Introduction

Human papillomavirus (HPV) is a common infecting agent, with certain high-risk types (HPV 16 and 18) that predispose men and women of varying ages to cancers such as anogenital (cervical, anal, and penile cancer) in addition to a subset of head and neck, particularly oropharyngeal, squamous cell carcinomas. The low-risk types (HPV 6 and 11) result in benign conditions such as warts [1-2]. The method of HPV transmission includes sexual intercourse and from the mother to her newborn during labor [3]. After infection, the virus could remain asymptomatic for years and the infection could clear on its own, but sometimes, the infection persists and progresses to cervical cancer. HPV infection is known to be the primary cause of cervical cancer in more than 70% of the total cervical cancer cases [4].

Cervical cancer is a major sequela of HPV infection, culpable for more than 311,000 deaths and 570,000 new patients per year [5]. Considering the fact that, at one point, the HPV burden accounted for 5.2% of the cancer load worldwide, the idea of vaccinating against HPV is equivalent to vaccinating against cancer [2], demonstrated by the decreasing prevalence of cervical cancer to 13,240 new cases per year, with a death toll of 4170 cases in a developed country such as USA [6]. While in contrast, 85% of the death toll from cervical cancer occurs in developing nations due to limited access to preventative measures [5]. HPV vaccination is estimated to decrease the incidence of cervical cancer by 42%-51% and reduce the mortality by 34%-45% [7].

Prophylactic vaccinations for high-risk HPV types are available, have been shown to be immunogenic, safe, and effective in the primary prevention of certain cancers and have an application window from 11-26 years [8].

A major issue faced against HPV vaccinations includes the lack of awareness about available options for vaccination and the negative attitude towards vaccination from the general public and healthcare professionals alike. Since medical students of today will be the practicing doctors of tomorrow, a need to assess their knowledge, attitude, and willingness to utilize HPV vaccination by including it as an essential addition to the vaccination regimen in children to protect them from preventable incidences of cancer [9].

While similar studies have been conducted worldwide, no studies have been reported on both male and female students from Riyadh, to assess the presumed advancement of the awareness of medical students from preclinical to clinical years. This study aims to fill this gap of knowledge and assess the level of knowledge between genders and their respective attitudes. We also attempt to gain an understanding of the possibility and the need for addressing the issue by including necessary changes in the medical school curriculum.

## Materials and methods

Ethical approval, study design, and instruments

This cross-sectional study was conducted at a University in Riyadh, Saudi Arabia. The institutional review board approved the research protocol for the present study (0036/05/2018-54). The data were collected using a validated and self-administrated questionnaire adapted and modified from a previous study, with a reliability study Cronbach alpha value of 0.7.

Sampling method and size

This study utilized the convenience-sampling method. The Raosoft calculator was used to calculate the sample size with a 5% margin of error and a 95% confidence level. Based on the above, the minimum sample size required for this study was 235, and 306 medical students eventually participated. The study population included male and female medical students from the first to the fifth years, with the first to third years being basic sciences years and the fourth and fifth being clinical sciences years. The study had an equal percentage of students representing each academic year (20%).

The only inclusion criterion was students studying the bachelor of medicine and surgery program at the university. The participants were given a study information sheet and an informed consent form, along with a survey questionnaire. Participants were informed that their anonymity and confidentiality would be maintained, and they could withdraw from the survey at any time.

Data analysis

The data analysis was performed using SPSS v 24 (IBM Corp., Armonk, NY, US) and R studio v 1.14 (Boston, US). Categorical data were summarized as counts and percentages. The questionnaire used was divided into four main sections:

1. Four questions to assess the participants’ demographics

2. Seventeen questions to assess the participants’ knowledge of the subject

3. Twelve questions to assess the participants’ attitudes toward HPV

4. A checkbox question to assess the source of information on HPV

Descriptive statistics

Descriptive statistics were performed to assess the number of respondents who answered each question correctly. The percentage of correct responses for each participant was calculated. The level of knowledge of the respondents was categorized based on the total score for the correct answers, with one mark for each correct answer, and the maximum score was 17. The categories included the high (12-17), moderate (6-11), and low (0-5) levels of knowledge. The mean knowledge score was also calculated. Attitudes towards HPV were assessed using a five-point Likert scale ranging from strongly agree to strongly disagree. Respondents’ attitudes were classified into positive (3.68-5), neutral (2.34-3.67), and negative (1-2.33) based on the total score.

Inferential statistics

Pearson’s correlation coefficient was used to determine the correlation between knowledge and the attitude toward HPV and cervical cancer. A p-value of less than 0.05 was considered statistically significant.

## Results

Descriptive statistics

Males accounted for 69% (n=211) of the study sample while females accounted for 31% (n=95). Most of the participants were aged 18-25 (n=289; 94.4%). All academic years were equally presented in the study sample (20% per each year), making the sample representative of the whole population and allowing for comparisons between groups. Most of the participants were unmarried (n=294, 96.1%), as shown in Table 1. The reliability of the questions addressing the attitude of participants was 0.75 (>0.7), which is considered acceptable.

**Table 1:** Descriptive statistics for the study sample (n=306)

.		
	Level	Count (%)
Gender (%)	Female	95 (31.0)
	Male	211 (69.0)
Age (%)	18-25	289 (94.4)
	>25	17 ( 5.6)
Academic year (%)	1st year	60 (19.6)
	2nd year	61 (19.9)
	3rd year	63 (20.6)
	4th year	61 (19.9)
	5th year	61 (19.9)
Marital status (%)	Single	294 (96.1)
	Married	6 ( 2.0)
	Other	6 ( 2.0)

Attitudes toward HPV

Results show that most participants were aware of the severity of HPV (92%). The majority (81%) were also aware of the fact that HPV is preventable and that the vaccine against HPV can prevent cervical cancer (83%). Responses were not consistent for Question 5, as only about half of the respondents (42%) thought that they were susceptible to HPV while the remaining did not (58%). Most participants thought that the HPV vaccine was safe (82%), and a high proportion (78%) thought that there was a lower risk associated with prevention (vaccination) than with infection.

Likewise, responses to Questions 7 through 12 show that participants have positive attitudes toward HPV prevention. The proportion of participants that agreed or strongly agreed to these questions exceeded 70%, except for Question 8 (vaccinating young people against HPV would not encourage them to become sexually active). Approximately half of the respondents agreed (55%) while the rest (45%) disagreed with the above statement.

The majority of the respondents showed a positive attitude towards HPV and were well aware of its risks (n = 177, 47.84%). The remaining respondents were neutral (n = 128, 41.83%) and only one respondent had a negative attitude toward HPV, as shown in Table 2.

**Table 2:** Attitude of medical students towards human papillomavirus HPV: human papillomavirus, SD: strongly disagree, D: disagree, N: neutral, A: agree, SA: strongly agree

	SD		D		N		A		SA		
	n	%	n	%	n	%	n	%		N	%
1. Cervical cancer is a severe disease.	1	0.3%	5	1.6%	34	11.1%	121	39.5%		145	47.4%
2. Cervical cancer is preventable.	2	0.7%	17	5.6%	77	25.2%	114	37.3%		96	31.4%
3. HPV vaccine is helpful to prevent cervical cancer.	4	1.3%	10	3.3%	74	24.2%	116	37.9%		102	33.3%
4. I am susceptible to HPV infection.	71	23.2%	60	19.6%	95	31.0%	48	15.7%		32	10.5%
5. HPV vaccine is safe.	1	0.3%	10	3.3%	90	29.4%	117	38.2%		88	28.8%
6. There is less risk involved in being vaccinated than having HPV infection.	8	2.6%	11	3.6%	95	31.0%	106	34.6%		86	28.1%
7. HPV vaccination will not lead to complicated sexual activities.	17	5.6%	30	9.8%	93	30.4%	98	32.0%		68	22.2%
8. Vaccinating young people against HPV would not encourage them to become sexually active.	32	10.5%	60	19.6%	93	30.4%	72	23.5%		49	16.0%
9. I would not want my children to be infected with HPV.	5	1.6%	12	3.9%	50	16.3%	52	17.0%		187	61.1%
10.Information on HPV helps me to decide whether my children should be vaccinated against HPV	4	1.3%	12	3.9%	74	24.2%	85	27.8%		131	42.8%
11.If my doctor thinks HPV vaccination is a good idea, I would have my children vaccinated	5	1.6%	16	5.2%	72	23.5%	95	31.0%		118	38.6%
12. I would have vaccinated against HPV if the vaccination was freely available.	5	1.6%	23	7.5%	97	31.7%	83	27.1%		98	32.0%

Knowledge regarding HPV

Results show that the percentage of correct answers ranged from 34.31% for Question 9 to 83.66% for Questions 2 and 11. Question 9 scored the lowest (HPV infection can resolve spontaneously), which indicates a serious lack of knowledge regarding HPV infection and management and can affect the attitude of medical students toward vaccination. Questions 2 (HPV infections are preventable) and 11 (HPV can cause genital warts) scored highest, with 83.66% of the participants answering each of these two questions correctly, as shown in Table 3.

**Table 3:** Knowledge of HPV and cervical cancer across participants (n=306) HPV: human papillomavirus, Q: question

	Question	N (%)
Q1	HPV can cause cervical cancer?	246 (80.4)
Q2	HPV infections are preventable?	256 (83.7)
Q3	Condom use can prevent HPV infection?	207 (67.6)
Q4	HPV is a sexually transmitted disease (STD)?	252 (82.4)
Q5	HPV infection is frequent?	172 (56.2)
Q6	HPV infection can last for years?	235 (76.8)
Q7	Cervical cancer is caused by persistent HPV infection?	242 (79.1)
Q8	HPV may infect both, men and women?	248 (81.0)
Q9	Most HPV infection resolves spontaneously?	105 (34.3)
Q10	HPV can infect you without symptoms?	219 (71.6)
Q11	HPV can cause genital warts?	256 (83.7)
Q12	HPV can cause other anogenital cancers (e.g. penis, anus and cervical)?	224 (73.2)
Q13	HPV vaccine prevents around 70% of cervical cancer?	229 (74.8)
Q14	Pap-smear can screen cervical cancer?	253 (82.7)
Q15	Pap-smear is very or relatively effective in screening cervical cancer?	239 (78.1)
Q16	Pap-smear should be done every 3 years?	199 (65.0)
Q17	Pap-smear can be done after the age of 21?	227 (74.2)
Score Mean (SD)		12.45 (3.30)
Knowledge (%)		
	Low	15 (4.9)
	Medium	70 (22.9)
	High	221 (72.2)

Knowledge regarding HPV was high among the participants, where only 15 (4.9%) had a low level of knowledge (score <5) and only 70 (22.9%) had moderate knowledge. A majority (n=221, 72.2%) had a high level of knowledge. The mean knowledge score was 12.45 (3.3).

Correlation between attitude and knowledge regarding HPV

There was a statistically significant correlation between attitudes towards HPV and knowledge regarding HPV (r=0.31, P <0.001 using Pearson's correlation), indicating that the participants who have a positive attitude toward HPV also have high knowledge regarding HPV and its risks.

Knowledge Source

**Figure 1:**
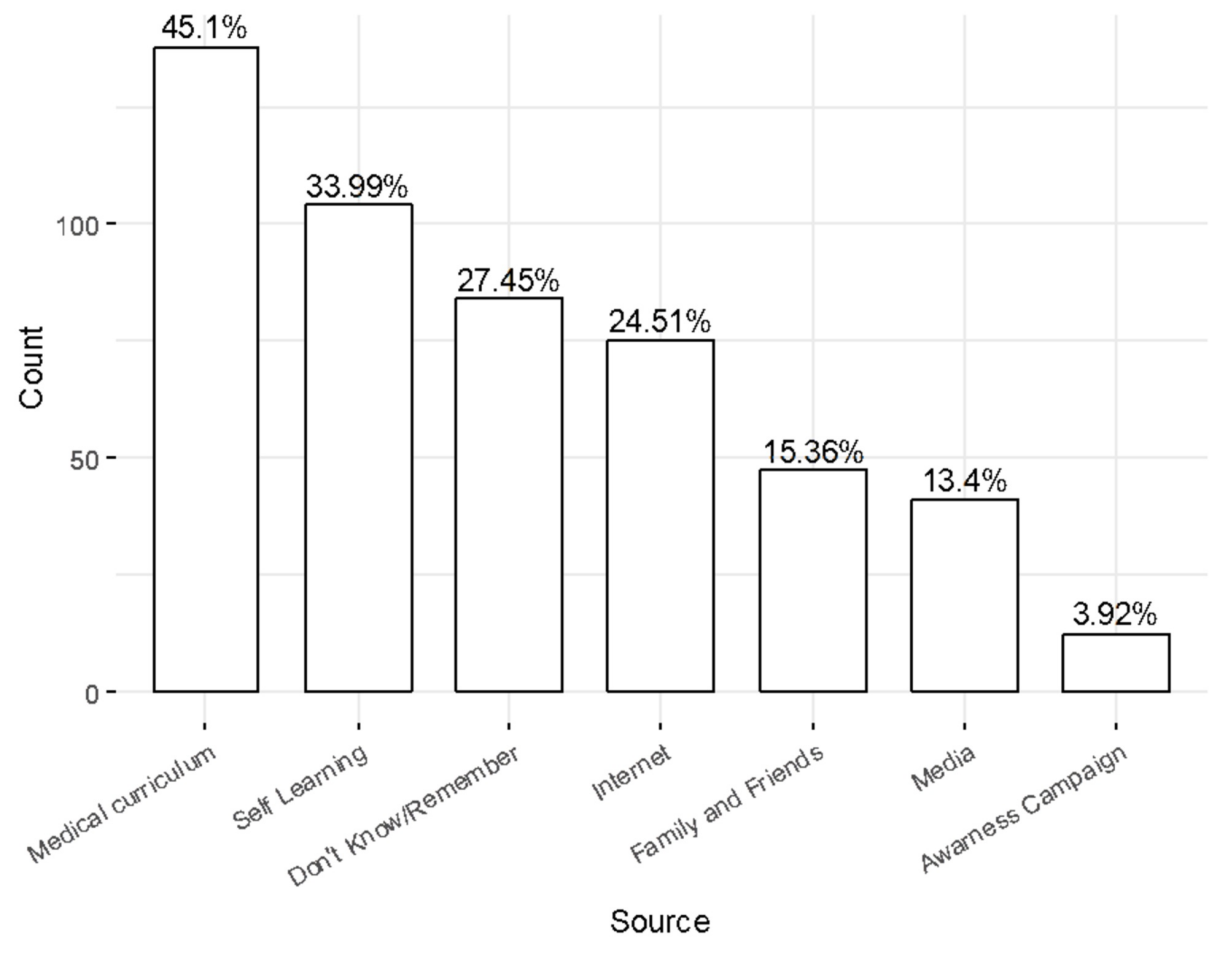
Source of information

The medical curriculum was the source of information for 45.1% of the students while self-learning was a source of knowledge for 33.99% of the students. However, 27.45% of the students did not remember the source and only 3.92% obtained their knowledge from awareness campaigns, as shown in Figure 1.

Interestingly, 82.61% of the students who mentioned the medical curriculum as their main source of information had a high level of knowledge in comparison to 61.7% of students who mentioned family and friends as their source of information on HPV. Likewise, 83.65% of students who mentioned self-learning as their main source of information had a high level of knowledge on the subject.

Association of demographics with knowledge score

The result shows that the mean knowledge score was significantly higher in females as compared to males (13.25 vs. 12.09, p<0.001). Neither age nor academic year was significantly associated with knowledge score (p=0.341 and 0.126, respectively), as shown in Table 4.

**Table 4:** Knowledge score based on age, gender, and academic year

		Knowledge score		
		Mean	SD	P
Gender	Female	13.25	1.99	<0.001*
	Male	12.09	3.69	
Age	18 - 25	12.49	3.30	0.341
	>25	11.71	3.41	
Academic year	1st	12.52	2.40	0.126
	2nd	12.49	3.31	
	3rd	12.54	3.59	
	4th	11.41	3.79	
	5th	13.28	3.05	

Knowledge regarding HPV based on gender

There was a statistically significant difference in the knowledge between males and females (P=0.028). None of the females had low knowledge as compared to 7.11% (n=15) of males, who scored low on knowledge of HPV. The proportion of females with moderate and high knowledge was also higher than the male students (24.2% and 75.8% vs. 22.3% and 70.6%, respectively).

## Discussion

The demographic data indicate a male majority in the sample population, attributed to the fact that the chosen academic institute had female students only from the first to third academic years. As results have elaborated, female students were more knowledgeable of HPV, with 75% of them having high knowledge and none having poor knowledge of HPV, which is in agreement with previously reported findings [1,10-11].

High level of knowledge in this student population is an important finding, as HPV vaccination specifically targets this age group [11]. As this age group is considered to be most susceptible to contracting serious malignant illness from HPV infection, it is all the more relevant, as the knowledge of female participants has a direct relationship with women's health.

With almost half of the students attributing the medical curriculum as their main source of knowledge regarding HPV, the finding also emphasizes the importance of including all-important information within the curriculum itself. Additionally, as one-third of the students cited self-learning as their source of knowledge, it is evident that the medical curriculum was not comprehensive and students felt the need to seek knowledge about the subject of HPV from other sources.

Although age and academic year were not relevant with respect to the extent of knowledge regarding HPV, there was an increment in knowledge among the students in the fifth year, suggesting that the exposure to health information of female patients and medical conditions in the obstetrician/gynecologist (OB/GYN) curriculum contributed to additional information on the subject. Overall, the level of knowledge was high among students, where 72.2% were highly knowledgeable regarding HPV, contradictory to the literature reports, where students were shown to have moderate to poor knowledge about HPV [11-14].

Our study indicates that there is an improvement in medical student's awareness concerning HPV in the surveyed Saudi university. According to our findings, better knowledge regarding HPV and its risks was associated with a better attitude toward HPV vaccination, which has been shown in other studies as well [11-14].

The student's attitude toward HPV vaccination informs us about the likelihood of these future doctors passing on the information regarding vaccination to their patients [15-17]. Based on this, we assessed our participant's attitude and found near-consensus about the preventability of HPV infections and the safety of the vaccination and an overall high level of awareness regarding HPV infections. However, one of the areas where their awareness was lacking included their own susceptibility to the infections, with 58% of the respondents believing that they were not in danger of contracting HPV.

Evidently, the greater majority proved to be inclined to vaccinate their kids, if their doctors thought it to be suitable, exemplifying the importance of a doctor's role in raising awareness. In addition, they themselves would get vaccinated if the vaccine was readily and freely available, which is also in agreement with findings previously reported in the literature [11-14].

The Malaysian government offers a free vaccination program, where all 13-year-old girls receive three doses of the vaccination [18]. Saudi Arabia offers the vaccination in its major hospital free of charge or for a designated fee [19]. This may act as an incentive for people to get vaccinated if they are informed and educated on the availability of the vaccine.

The study shows that the respondents were overall very knowledgeable, with 83.66% knowing that HPV is preventable and it leads to genital warts. Of the respondents, 80.4% were aware that HPV could cause cervical cancer, which is clinically important, as 89% of cervical cancer cases in Saudi Arabia are linked to HPV infection, with a prevalence rate that is comparable to the international prevalence rate [19].

Almost half of the surveyed students did not know that HPV infection is frequent, and two-thirds of the respondents were unaware that HPV infection could resolve spontaneously, indicating a lack of understanding of the duration and frequency of HPV infection and its tendency to regress. The correlation between the level of knowledge and the source of information proved that students who were more inclined toward self-learning had a higher level of knowledge (83.65%), closely followed by those who learned from their medical curriculum (82.61%), emphasizing the need to draft a medical curriculum that incorporates all the necessary information for the students. Students who cited family, friends, and other people from their social circles as a source of knowledge proved to be the least knowledgeable, which exemplifies the need to raise awareness within the society and among medical students with regards to HPV virus and infections.

Limitations

One of the limitations of the present study is that it has been conducted at a single medical college. This college only recently started admitting female students and at the time of this study, the medical college had female students only up to the third academic year.

## Conclusions

In conclusion, the mean knowledge score regarding HPV is higher in female medical students as compared to males. There was no relationship between age and academic year regarding the extent of knowledge about HPV. A better attitude towards HPV correlates with better knowledge of HPV and its risks.

Early improvements in education can help future doctors treat and decrease the incidence of HPV by imparting comprehensive information on the risks of HPV transmission as well as preventive measures to all years of the medical school curriculum, as students largely depend on their curriculum as a primary source of information on HPV. More effective awareness programs need to be introduced to educate the general public about the risks associated with HPV and the benefits of the HPV vaccine.
